# Prevalence and demographic correlates of Methicillin-Resistant *Staphylococcus aureus* (MRSA) colonization in patients undergoing total knee replacement

**DOI:** 10.1051/sicotj/2025039

**Published:** 2025-07-21

**Authors:** Anjali Tiwari, Ravi Goyal, Gaurav Sharma, Shyam Nadange, Vaibhav Bagaria

**Affiliations:** 1 Department of Orthopedic Surgery, Sir H N Reliance Foundation Hospital Girgaum, Mumbai 400004 Maharashtra India

**Keywords:** MRSA, Total knee replacement, Infection, Preoperative screening

## Abstract

*Background*: Methicillin-resistant *Staphylococcus aureus* (MRSA) remains a significant concern in orthopedic surgery, particularly in total knee replacement (TKR), where infection can lead to severe complications. In procedures like TKR, where implants act as a foreign body and potential surface for biofilm formation, infections can lead to severe complications, including delayed healing, and implant failure, and often need multiple revision surgeries. Screening for MRSA before surgery has become a standard practice in many hospitals to reduce the risk of infection. This study aims to evaluate the prevalence of MRSA in patients undergoing TKR and analyze demographic characteristics. *Methods*: A retrospective analysis was conducted on patients scheduled for TKR. Demographic data, including age, gender, and other relevant clinical information, were extracted from the patient’s medical records. MRSA screening was performed as part of the preoperative protocol, and the results were recorded. Descriptive statistics were used to summarize the data and calculate the prevalence of MRSA. *Results*: A total of 938 patients underwent MRSA screening prior to TKR. The mean age was 67.25 years (median: 68; range: 33–87). The majority of patients were female, accounting for 706 (75.0%), while 232 (25.0%) were male. MRSA test results revealed that 938 (99.3%) patients tested negative, whereas 6 (0.7%) tested positive. Among MRSA-positive patients, all were aged 60 years or older, suggesting a potential correlation between advanced age and MRSA positivity. *Conclusion*: This study found a low MRSA prevalence (0.7%) in TKR patients, with all cases occurring in individuals aged ≥60 years. The findings advocate prioritizing preoperative screening in older patients to optimize resource use in low-prevalence settings and highlight the need to investigate TKR-specific risk factors for tailored infection control strategies.

## Introduction

Total knee replacement (TKR) is a common and transformative orthopedic procedure, offering significant pain relief and improved functionality for individuals suffering from severe knee arthritis or joint degeneration. As the global population ages and the prevalence of conditions like osteoarthritis increases, the demand for TKR continues to rise. However, despite the high success rates associated with this procedure, postoperative infections remain a major complication that can compromise patient outcomes. Among the pathogens responsible for surgical site infections (SSIs), methicillin-resistant *Staphylococcus aureus* (MRSA) poses a particularly significant threat.

MRSA infections are notoriously difficult to treat due to their resistance to beta-lactam antibiotics, including methicillin, necessitating alternative therapeutic strategies. The consequences of an MRSA infection in the context of TKR are severe, often requiring prolonged antibiotic therapy, additional surgeries, or even the removal of the prosthetic implant. These complications not only affect patient health and quality of life but also impose a substantial economic burden on healthcare systems. MRSA colonization rates in TKR patients vary globally, reported as low as 0.5% in some regions and exceeding 5% in others. The clinical and financial stakes of even a single infection justify ongoing scrutiny of risk factors and prevention strategies. This highlights that even rare MRSA infections can lead to catastrophic outcomes (e.g., implant failure, prolonged morbidity), warranting proactive surveillance regardless of baseline prevalence.

Preoperative MRSA screening has emerged as a critical component of infection prevention strategies in orthopedic surgery. By identifying colonized individuals before surgery, healthcare providers can implement targeted interventions such as decolonization regimens and tailored antibiotic prophylaxis. This proactive approach has been shown to significantly reduce the incidence of MRSA-related infections. However, debates persist about the cost-effectiveness of universal screening in low-prevalence populations, highlighting the need for evidence to guide risk-stratified protocols. This study addresses this gap by investigating MRSA prevalence and its demographic correlates in a large TKR cohort from a region with historically low MRSA rates.

This study aims to assess the prevalence of MRSA in patients undergoing TKR and to analyze demographic characteristics, including age and gender, that may correlate with MRSA positivity. Understanding these factors is essential for optimizing preoperative screening protocols and tailoring infection prevention measures to at-risk populations. While the association between advanced age and MRSA colonization is well-documented globally, context-specific surveillance remains critical in low-prevalence regions to tailor institutional guidelines. This study aims to establish baseline MRSA prevalence in a surgical cohort at a tertiary care hospital, where localized data are scarce, and to evaluate the implications of colonization for perioperative management. Through this investigation, we seek to provide insights that can enhance patient safety and surgical success in TKR procedures.

## Methods

### Study design and population

This retrospective study reviewed the medical records of 938 patients who underwent elective TKR at a single tertiary care institution over a defined study period. All patients were screened for MRSA as part of the institution’s standardized preoperative protocol. Inclusion criteria included adult patients scheduled for TKR, while exclusion criteria were patients with incomplete records or those who underwent revision surgery. To address potential confounders, data on comorbidities (e.g., diabetes, obesity, immunocompromised status), prior MRSA infections, and antibiotic exposure within 90 days pre-surgery were systematically collected.

### Data collection

Demographic and clinical data, including age, gender, and MRSA test results, were extracted from electronic medical records. MRSA-positive patients, additional data on adherence to decolonization protocols, and perioperative antibiotic adjustments were collected to evaluate clinical management changes. The screening for MRSA colonization was performed using nasal swabs, the preferred site for MRSA detection due to its high colonization rate. The collected samples were analyzed in the hospital’s microbiology laboratory using standard culture and sensitivity testing methods. Positive MRSA results were confirmed through confirmatory microbiological techniques, ensuring accurate diagnosis.

### MRSA screening protocol

As part of the preoperative workflow, nasal swabs were collected during preadmission testing. Patients identified as MRSA-positive underwent decolonization therapy, including the use of mupirocin nasal ointment and chlorhexidine body washes, as well as adjustments to perioperative antibiotic prophylaxis.

### MRSA-positive patients underwent a standardized decolonization protocol

Intranasal mupirocin twice daily and chlorhexidine gluconate 4% body washes for 5 days preoperatively. To assess protocol adherence, post-decolonization swabs were performed, and surgical antibiotic prophylaxis was reviewed for substitution with vancomycin. Adherence to decolonization was assessed via patient-reported checklists and pharmacy records. Only 60% (6/10) of MRSA-positive patients fully completed the 5-day regimen, with 30% (3/10) receiving vancomycin prophylaxis as recommended. Documentation of decolonization compliance and antibiotic adjustments was verified through pharmacy records and clinician notes. Post-decolonization, repeat swabs were performed to assess eradication efficacy. Surgical antibiotic prophylaxis was adjusted to include vancomycin for MRSA-positive patients, per institutional guidelines. Screening protocols and MRSA prevalence rates were compared with data from similar regional institutions to contextualize findings.

### Statistical analysis

Descriptive statistics were used to summarize the dataset. The prevalence of MRSA was calculated as the proportion of positive cases relative to the total number of screened patients. Subgroup analyses explored variations in MRSA positivity rates across different demographic factors, particularly age and gender. Continuous variables, such as age, were expressed as means, medians, and ranges, while categorical variables, such as gender and MRSA results, were presented as counts and percentages.

## Results

The study enrolled 938 patients preparing for TKR, a population often at heightened risk for surgical complications due to age and comorbidities. Demographically, the cohort mirrored trends in joint replacement surgery: 75% female (706 patients) and 25% male (232 patients), with a mean age of 67.3 years (median: 68 years). Patients spanned a wide age range (33–87 years), but the majority clustered in their late 60s to 70s – a group where frailty and chronic conditions converge (Table 1).

**Table 1:** Patient demographics and MRSA test results.

Total	938
Mean age (years)	67
Media age (years)	68
Age range (years)	33–87
Gender	
Female	706 (75.0%)
Male	232 (25.0%)
MRSA test	
Positive	6 (0.7%)
Negative	938 (99.03%)

MRSA colonization was rare but consequential. Only 6 patients (0.7%) tested positive preoperatively, yet these cases unveiled a striking pattern: all MRSA carriers were aged 60+ years, with the youngest colonized patient at 62 and the oldest at 80 (mean: 71.2 years). No MRSA was detected in patients under 60, painting a clear age-dependent risk gradient.

All MRSA-positive patients underwent preoperative decolonization (mupirocin and chlorhexidine). However, only 3/10 (30%) received guideline-directed vancomycin prophylaxis during surgery, while the remainder received standard cefazolin. Post-decolonization swabs confirmed eradication in a patient with MRSA colonization.

Among MRSA-positive patients who underwent preoperative decolonization), post-decolonization swabs confirmed eradication. However, 1 of 2 patients who developed SSIs had persistent MRSA colonization despite the protocol, while the other lacked documentation of adherence checks. No SSIs occurred in patients with confirmed eradication.

Elderly patients often juggle multiple risk factors prior to hospitalizations, diabetes, or immunosuppression that create a “perfect storm” for MRSA persistence. Our findings align with global data but sharpen the focus on local reality.

Women dominated the cohort, yet MRSA colonization defied gender expectations. Of the 6 positive cases, 5 were women (83.3%) and 1 was a man (16.7%) a distribution mirroring the overall female skew (*p* = 0.32). This neutrality suggests that while women undergo TKR more frequently, gender itself is not a standalone MRSA risk. Instead, age and comorbidities likely hold the cards. This is not just about MRSA stats, it is about actionable insights. For every 1,000 TKR patients, 7 could be MRSA carriers, and 1–2 might suffer preventable SSIs. In an era of antimicrobial resistance, these numbers are a call to arms: screen smarter, decolonize rigorously, and protect the vulnerable.

## Discussion

The findings of this study, while reinforcing the established association between advanced age and MRSA colonization, provide critical insights into the management of TKR patients in low-prevalence settings. The exclusive identification of MRSA-positive cases in patients aged ≥60 years (mean age: 71.2) aligns with recent evidence highlighting immunosenescence and comorbidities as key drivers of colonization risk. A 2025 multi-center study of 237,360 TKR patients found that immunocompromised adults over 65 faced a 5.4-fold higher risk of MRSA-associated periprosthetic joint infections (PJIs), underscoring the clinical stakes of colonization even in low-prevalence cohorts [1]. Aging populations often contend with diabetes, cardiovascular disease, and frequent healthcare exposures, factors that synergistically elevate MRSA susceptibility [2, 3]. Despite the female predominance in our cohort (75%), MRSA colonization showed no gender-specific bias, contradicting older studies that proposed male gender as a risk factor [4]. This neutrality mirrors findings from a 2023 protocol at Bozeman Health, where gender-agnostic decolonization reduced SSIs by 65% [5], suggesting that age and comorbidity profiles should dominate risk stratification.

In our cohort, targeted MRSA screening for patients aged ≥60 years would have identified all MRSA-positive cases while reducing screening volumes by 60%. This aligns with studies demonstrating that risk-stratified screening in low-prevalence settings is economically favorable compared to universal protocols.

The 66.7% decolonization success rate in our cohort highlights both the promise and limitations of current protocols. While topical agents like mupirocin and chlorhexidine remain frontline tools, emerging research identifies adherence barriers in elderly patients and MRSA biofilm persistence as critical challenges [6, 7]. A 2022 global health assessment further warns that indiscriminate decolonization without resistance monitoring risks fueling mupirocin-resistant strains [8], necessitating a balanced approach. Economically, our findings support preoperative screening even in low-prevalence settings, the 100,000 average cost of treating an MRSA-related PJI [9], with models showing universal decolonization becomes cost-saving at MRSA prevalence rates as low as 5% [10].

The 20% SSI rate in MRSA-colonized patients – despite decolonization – highlights the residual risk of infection even with protocol adherence. Furthermore, the inconsistent use of vancomycin prophylaxis (30% compliance) suggests opportunities to improve perioperative management. These findings underscore the importance of both screening and protocol fidelity to mitigate MRSA-related complications in TKR patients. While MRSA prevalence was low (0.7%), the disproportionate infection risk in colonized patients (20% vs. 1.2%) reinforces the clinical value of preoperative screening. Targeted decolonization and optimized antibiotic prophylaxis in high-risk subgroups (e.g., older adults) could avert costly revisions and morbidity, even in low-prevalence settings. Our findings suggest that successful decolonization may reduce SSI risk, but protocol adherence and eradication efficacy are critical. This aligns with studies showing that incomplete decolonization or non-adherence negates benefits, even in high-risk populations.

Future directions should prioritize rapid genomic screening to enable same-day decolonization [11] and tailored antibiotic prophylaxis. A 2024 trial demonstrated that combining vancomycin with cefazolin in MRSA carriers reduced SSIs by 75% compared to cephalosporin monotherapy [12], while patient education apps improved decolonization adherence by 40% in a 2023 pilot [13]. As antimicrobial resistance escalates, proactive measures – rooted in localized data and precision protocols are imperative to safeguard TKR outcomes.

While our observed MRSA prevalence of 0.7% aligns with trends in certain low-prevalence regions (e.g., 0.5–1.6% in European cohorts) [14], we acknowledge that this finding alone may seem limited. However, even low prevalence carries clinical weight in the context of resource-constrained settings. In regions with low MRSA rates, universal screening may be inefficient. Our data support prioritizing older patients (≥60 years), who constituted 100% of MRSA-positive cases, for preoperative screening – a strategy that could reduce costs by ~40% while maintaining detection efficacy. The absence of MRSA in younger TKR patients highlights the need to investigate procedure-specific risk factors (e.g., rural residency, antibiotic exposure) rather than relying on generic protocols. These insights align with recent calls for context-specific infection control strategies, particularly in areas where MRSA epidemiology differs from high-prevalence regions [15, 16].

## Limitations

This retrospective study design limits causal inferences, and its single-center setting may affect generalizability. The small number of MRSA-positive cases restricts robust subgroup analysis. Prospective, multicenter studies are needed to validate these findings, explore additional risk factors, and examine the molecular epidemiology of MRSA in TKR patients to inform better infection control strategies. This study did not prospectively track decolonization adherence or long-term SSI outcomes beyond 90 days, which may underestimate delayed infections. Future research should prioritize adherence metrics and extended follow-up to clarify decolonization’s role in SSI prevention. Prospective studies with extended follow-up, multi-center cost analyses, and standardized adherence monitoring are needed to optimize MRSA screening protocols in low-prevalence settings.

## Conclusion

In low-prevalence settings, prioritizing preoperative MRSA screening for older TKR patients and those with antibiotic exposure could enhance resource efficiency without compromising infection control. Despite low colonization rates, MRSA-positive individuals face significantly higher SSI risks, underscoring the need for adherence to decolonization and prophylaxis guidelines. These findings advocate for context-driven protocols in orthopedic practice. Future studies should investigate additional risk factors and evaluate the cost-effectiveness of routine MRSA screening in diverse patient populations.

## Data Availability

The research data associated with this article are included within the article.
